# Increased EGFR Phosphorylation Correlates with Higher Programmed Death Ligand-1 Expression: Analysis of TKI-Resistant Lung Cancer Cell Lines

**DOI:** 10.1155/2017/7694202

**Published:** 2017-10-08

**Authors:** Kenichi Suda, Leslie Rozeboom, Koh Furugaki, Hui Yu, Mary Ann C. Melnick, Kim Ellison, Christopher J. Rivard, Katerina Politi, Tetsuya Mitsudomi, Fred R. Hirsch

**Affiliations:** ^1^Division of Medical Oncology, University of Colorado Anschutz Medical Campus, 12801 E. 17th Ave. RC-1 South, Rm 8402J, Aurora, CO 80045, USA; ^2^Division of Thoracic Surgery, Department of Surgery, Kindai University Faculty of Medicine, 377-2 Ohno-higashi, Osaka-Sayama 589-0014, Japan; ^3^Product Research Department, Kamakura Research Laboratories, Chugai Pharmaceutical, 200 Kajiwara, Kamakura 247-8530, Japan; ^4^Department of Pathology, Yale University School of Medicine, 310 Cedar St. LH 108, New Haven, CT 06520, USA

## Abstract

Despite the recent development of immunotherapies that target programmed death-1 (PD-1) or programmed death ligand-1 (PD-L1) in non-small cell lung cancer (NSCLC) treatment, these therapies are less effective in NSCLC patients with* epidermal growth factor receptor (EGFR)* mutations. However, the molecular mechanisms underlying this lower efficacy of immunotherapies in* EGFR* mutant lung cancers are still unclear. In this study, we analyzed PD-L1 protein expression in lung cancer cell lines with* EGFR* mutations prior to and after acquisition of resistance to EGFR tyrosine kinase inhibitors (TKIs). We found that parental lung cancer cell lines harboring* EGFR* mutations showed negative (PC9 and H3255 cells) and positive (HCC827 cells) staining for PD-L1 by immunohistochemistry. Comparing PD-L1 expression between EGFR-TKI resistant cell lines and their parental cells, we found that increased phosphorylation of EGFR was related to increased expression of PD-L1. Increased phosphorylation of EGFR was accompanied by the T790M secondary mutation. Acquired resistance cells with* MET* amplification or* EGFR* loss both showed decreased phosphorylation of EGFR and decreased PD-L1 expression. Our results indicate that lung cancer cell lines with* EGFR* mutations (parental cells) do not harbor high PD-L1 protein expression. In addition, EGFR phosphorylation affects PD-L1 expression after acquisition of resistance to EGFR-TKIs.

## 1. Introduction 

Activating mutations in the* epidermal growth factor receptor (EGFR)* gene define one of the most common molecular subtypes of non-small cell lung cancers [1]. EGFR tyrosine kinase inhibitor (TKI) monotherapies (gefitinib, erlotinib, or afatinib) are the first choice for these patients [1]; however, acquisition of resistance to these TKIs is almost inevitable after an average of 1 year [2]. A variety of resistance mechanisms have been identified including T790 M mutation,* MET* or* ERBB2* gene amplification, small cell lung cancer transformation, and epithelial to mesenchymal transition (EMT) [2].

Osimertinib, a 3rd generation EGFR-TKI, is the appropriate second-line drug after acquisition of resistance to gefitinib, erlotinib, or afatinib if a rebiopsied resistant tumor proves the presence of an* EGFR* T790M secondary mutation [3]. However, cytotoxic chemotherapies are still the standard of care as second-line drugs for patients who do not have the* EGFR* T790M mutation [4]. Recent development of immunotherapies that target programmed death ligand-1 (PD-L1) or programmed death-1 (PD-1) has shown dramatic success in some lung cancer patients [5]. However, these immune-checkpoints inhibitors have shown poorer response rates and outcomes in patients with* EGFR* mutations compared to those with* EGFR* wild-type tumors [6, 7].

PD-L1 protein expression has been pursued as a predictive marker for current immunotherapies. To elucidate the underlying mechanisms of this reduced effectiveness for immunotherapies in lung cancer patients with* EGFR* mutations, we performed the current study to analyze PD-L1 protein expression status, using the FDA approved detection kit system, before and after the acquisition of resistance to EGFR-TKIs in established cell lines harboring* EGFR* mutations.

## 2. Materials and Methods

### 2.1. Cell Lines, Reagents, and Generation of* In Vitro* Resistant Cell Lines

Human lung cancer cell lines used in this study were from the established collections in our labs or as reported in our previous studies [8–10]. PC-9 erlotinib resistant cells were established from PC-9 cells by stepwise exposure to erlotinib from 0.005 *μ*M to 5 *μ*M for about 4 months, and the clone named PC-9 ER clone 5 was isolated with PicoPipet (Nepa Gene, Chiba, Japan). Cells were cultured in RPMI1640 medium supplemented with 10% fetal bovine serum (FBS) and 1x penicillin/streptomycin solution (Mediatech, Inc., Manassas, VA) at 37°C in a humidified tissue culture incubator with 5% CO_2_. All experiments using acquired resistance cells were performed following the removal of drug exposure to avoid the direct effects of drugs on PD-L1 expression. IFN-gamma (Cell Signaling Technology, Dancers, MA) stimulation for 24 hours was performed to mimic an immune cell interaction.

### 2.2. Immunohistochemistry (IHC) Analysis

Formalin-fixed paraffin-embedded (FFPE) blocks were prepared from cell pellets to perform IHC analysis as previously described [11]. Briefly, cultured cells were gently harvested using Accutase® (Innovative Cell Technologies, Inc., San Diego, CA) and fixed with alcoholic formalin solution for 24 hours. Fixed cells were mixed with melted agarose solution, allowed to solidify, placed in the cassette, and submerged in 70% ethanol. Paraffin-embedding of the agarose cell pellet was performed at our pathology core lab. Antibody against PD-L1 was purchased from Dako (22C3 pharmDx, Dako–Agilent Technologies, Carpinteria, CA). Staining was performed on a Dako Link 48 Auto-Stainer. PD-L1 staining was assessed using the *H*-score assessment.

### 2.3. Western Blot Analysis

All antibodies were purchased from Cell Signaling Technology (Danvers, MA). Total cell lysates were prepared, and immunoblotting was conducted as described previously [11]. Briefly, cells were cultured until subconfluent, rinsed with phosphate-buffered saline (PBS), lysed in sodium dodecyl sulfate (SDS) sample buffer, and homogenized. The total cell lysate (10 *μ*g) was subjected to SDS polyacrylamide gel electrophoresis (PAGE) and transferred to Immobilon-P polyvinylidene difluoride (PVDF) membranes (Millipore, Bedford, MA). After blocking with 5% nonfat dry milk, membranes were incubated with primary antibodies, washed with PBS, and reacted with secondary antibodies (Cell Signaling Technology), and signals visualized using ECL reagent (Clarity, Bio-Rad, Hercules, CA) and film or detected by an ImageQuant Imager (GE Healthcare Bio-Sciences, Tokyo, Japan).

### 2.4. Flow Cytometry

PC-9 and PC-9 ER clone 5 cells were stained with the mouse monoclonal BV421-conjugated antibody to human PD-L1 (BD Biosciences, San Jose, CA) for flow cytometry analysis with the BD LSRFortessa cell analyzer and the BD FACSDiva software (BD Biosciences).

## 3. Results

### 3.1. PD-L1 Expression in Lung Cancer Cell Lines with* EGFR* Mutation

**Figure 1:**
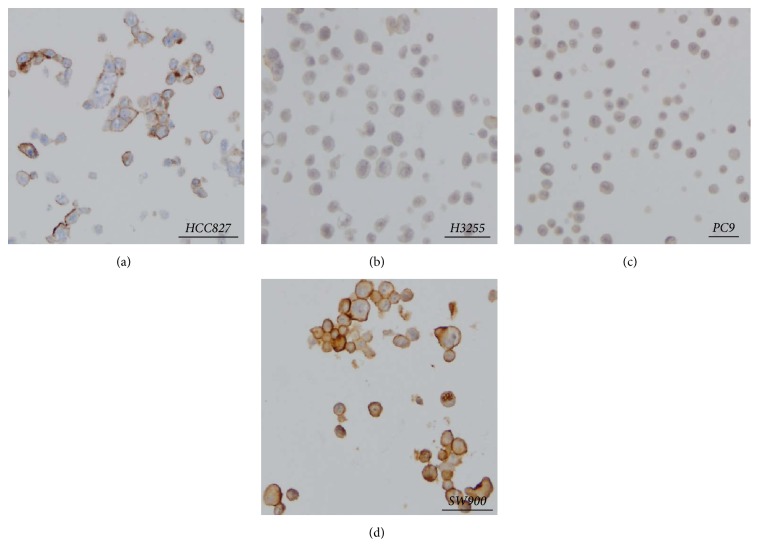
PD-L1 expression in parental lung cancer cell lines with* EGFR* mutations by IHC (Dako 22C3 antibody). (a) HCC827 showed positive staining (*H*-score: 145). (b) and (c) H3255 and PC-9 cells demonstrate negative staining. (d) SW900 lung squamous cells with positive staining. Images at 20x, captured with a Olympus DP71.

Initially, we screened for PD-L1 expression in parental lung cancer cell lines by IHC using the Dako 22C3 antibody. The efficacy of the 22C3 antibody was recently demonstrated in clinical trials [12, 13], and the analytical performance seems similar to two other clinically used PD-L1 antibodies (Dako 28-8 and Ventana SP- 263 [14]). As shown in Figure 1, PC-9 cells (del E746_A750) and H3255 cells (L858R) were negative for PD-L1 IHC, and HCC827 cells (del E746_A750) showed positive expression for PD-L1 membrane staining (*H*-score: 145) that is weaker than that observed in other non-small cell lung cancer cell lines such as SW900, a lung squamous cancer cell line (*H*-score: 210). These results indicate that lung cancer cell lines harboring* EGFR* mutation do not have high PD-L1 protein expression prior to EGFR-TKI exposure.

### 3.2. EGFR Phosphorylation Levels after Acquisition of Resistance to TKIs Is Related to PD-L1 Expression Status

**Figure 2:**
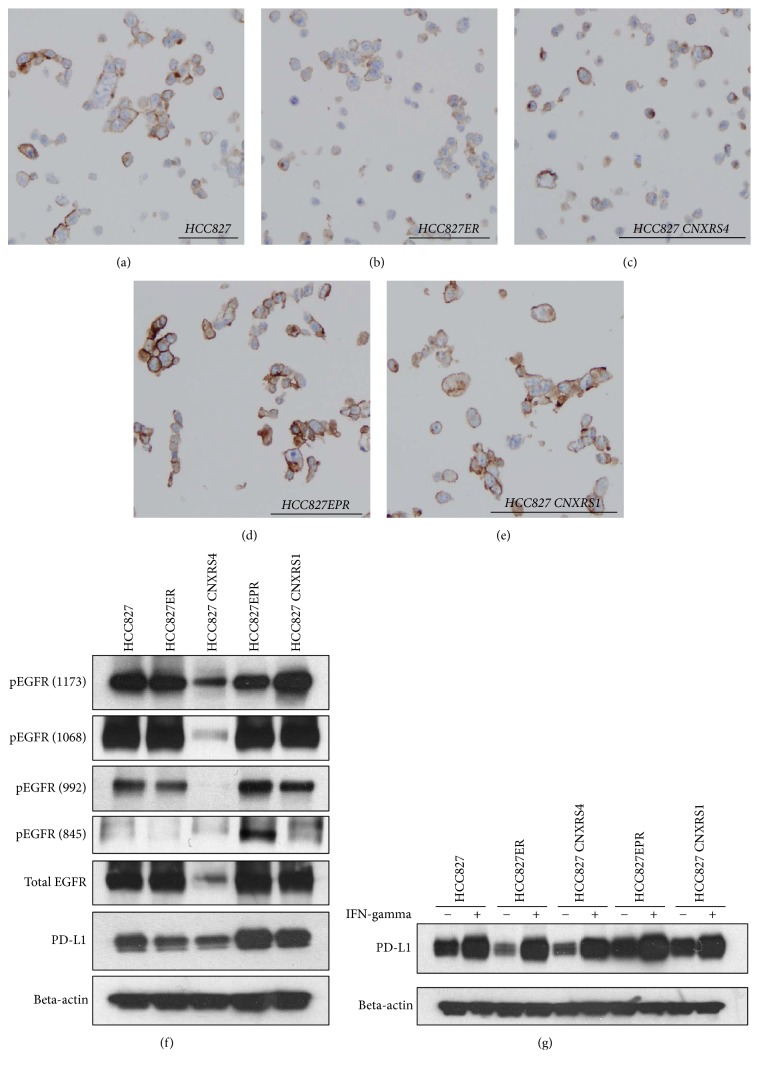
PD-L1 expression in parental HCC827 cells and their EGFR-TKI resistant daughter cells. (a)–(e) IHC staining for PD-L1 (Dako 22C3 antibody) in each cell line, showing decreased PD-L1 expression in HCC827ER and HCC827 CNXRS4 cells and increased PD-L1 expression in HCC827EPR and CNXR S1 cells. (f) Western blot analysis for PD-L1 and phosphorylation of EGFR. Beta-actin was used as loading control. (g) The effect of IFN-gamma exposure (100 U/ml, 24 hrs) for PD-L1 expression in HCC827 and acquired resistance daughter cell lines.

Initial evaluation for PD-L1 protein expression changes focused on comparisons between the parental HCC827 cells and a group of daughter cell lines with acquired resistance to EGFR-TKIs. Each daughter cell line exhibits different type of resistance mechanisms including* MET* gene amplification (HCC827ER), T790M mutation (HCC827EPR),* MET* gene amplification together with T790M mutation (HCC827 CNXR S1), and* MET* gene amplification with* EGFR* loss (HCC827 CNXR S4). As shown in Figures 2(a)–2(e), HCC827 daughter cells that have acquired resistance to EGFR-TKIs demonstrated various PD-L1 expression patterns including slightly decreased PD-L1 expression in HCC827ER and HCC827CNXR S4 cells (*H*-scores: 125 and 120, resp.) compared with parental cells (*H*-score: 145). Meanwhile, both of T790M positive lines (HCC827EPR and HCC827 CNXR S1 cells) had higher PD-L1 expression (*H*-scores: 220 and 190, resp.) compared with parental cells. These results were confirmed using western blot analysis (Figure 2(f)).

Because previous studies have observed a relationship between PD-L1 expression and EGFR activation in lung cancers (but no data regarding EGFR-TKI resistance and PD-L1 expression) [15–18], we compared EGFR phosphorylation status between these cells. We observed that the acquired resistance cells with PD-L1 downregulation have decreased phosphorylation of EGFR (Y992 in HCC827ER cells and all EGFR phosphorylation sites in HCC827CNXRS4 cells). On the other hand, resistant cells with upregulated PD-L1 have increased phosphorylation of EGFR (Y992 and Y845 in HCC827EPR cells and Y1173 in HCC827CNXRS1 cells).

**Figure 3:**
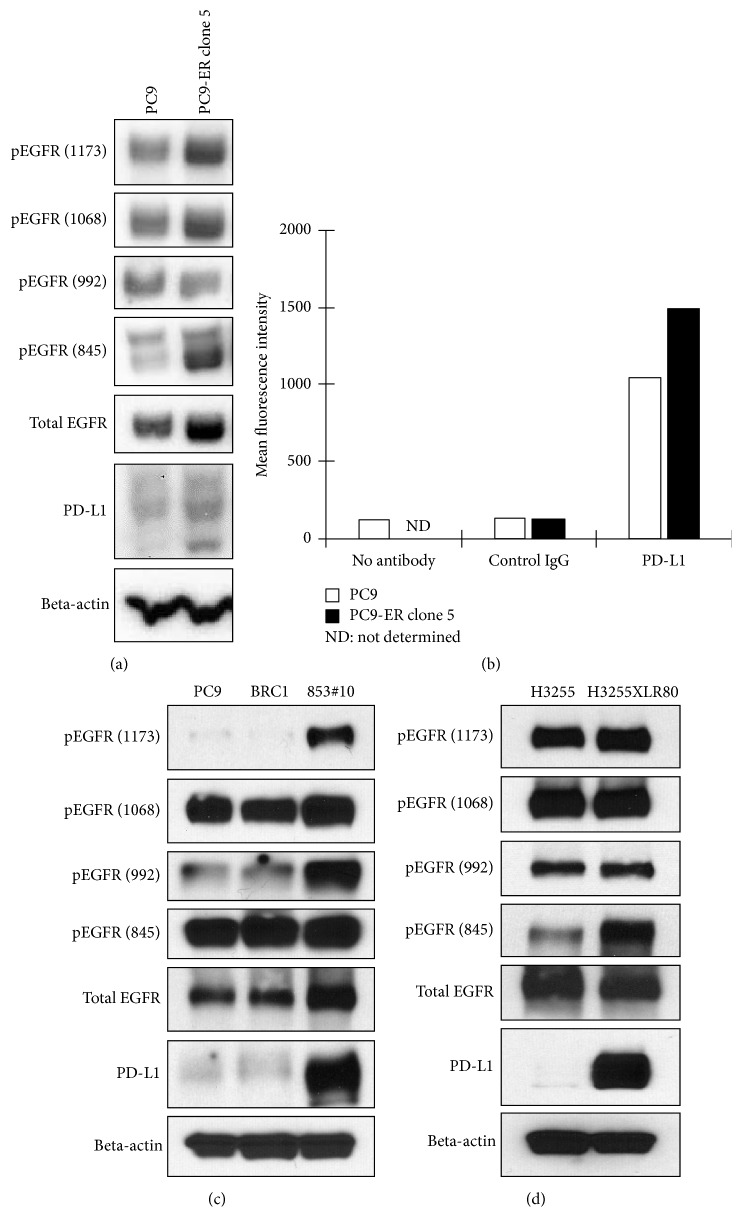
PD-L1 expression and EGFR phosphorylation status in H3255 and PC-9 cells that acquired resistance to EGFR-TKIs. (a) and (b) PC-9 ER clone 5 showed moderate increase of p-EGFRs together with slight increase of PD-L1 expression by western blotting (a) and flowcytometry (b). (c) The other series of PC-9 daughter cell lines resistant to EGFR-TKIs. BRC1 cells showed similar phosphorylation status of EGFR and PD-L1 compared with parental cells, while 853#10 showed dramatic increase of p-EGFR (Y992 and Y1173) and PD-L1. (d) H3255XLR80 also showed dramatic increase of p-EGFR (Y845) and PD-L1.

To generalize our findings in the HCC827 series, we employed two other cell lines (PC-9 and H3255 cells) that developed acquired resistance to EGFR-TKIs via T790  mutation (PC-9ER clone 5, BRC1, 853#10, and H3255XLR80 cells). As shown in Figure 3, three out of four cell lines had higher PD-L1 expression together with increased phosphorylation of EGFR compared to their parental cells. These data also indicated that no single phosphorylation site of EGFR is most responsible for increased PD-L1 expression. Only BRC1 cells showed similar EGFR phosphorylation and PD-L1 expression status compared with parental PC-9 cells.

### 3.3. Effect of IFN-Gamma Treatment on Parental and Resistant Cells

It has been established that cytokine signaling from the surrounding tumor microenvironment regulates PD-L1 expression in tumor cells [19]. To mimic an immune cell interaction, we treated HCC827 and their resistant daughter cell lines with IFN-gamma and measured PD-L1 expression. We observed that IFN-gamma treatment induced significant amount of PD-L1 protein in all cells examined (Figure 2(g)).

## 4. Discussion

In this study, we observed that cell lines with acquired EGFR-TKI resistance that harbor increased phosphorylation of EGFR, at any tyrosine residue, showed upregulation of PD-L1 protein expression. This finding is consistent with previous reports, which have observed that EGF stimulation upregulates PD-L1 expression or EGFR inhibition downregulates PD-L1 expression [15–18]. Each phosphorylation site of EGFR provides a binding surface for different substrate proteins, for example, GRB2 adaptor protein – Y1068 [20] or the SH2 domain of PLCgamma – Y992 [21]. However, all major downstream signaling pathways of EGFR, such as AKT serine/threonine kinase (AKT) – mechanistic target of rapamycin (mTOR) pathway [22], Janus kinase (JAK) – signal transducer and activator of transcription (STAT) pathway [23], or mitogen-activated protein kinase 1 (MAPK1) pathway [17], are reported to induce PD-L1 expression. Therefore, it would be reasonable that increased phosphorylation of any tyrosine residue of the EGFR is correlated with increased expression of PD-L1.

Our results may also provide for a possible explanation for lower efficacy of current immunotherapies in lung cancer patients with an* EGFR* mutation. We observed that PD-L1 protein expression is not high in parental cells with* EGFR* mutation, and the PD-L1 expression decreased when cells developed resistance to EGFR-TKIs by a non-T790M mediated resistance mechanism. In our previous study, we also found that EMT, another non-T790M mediated resistance mechanism to EGFR-TKIs, decreased PD-L1 expression in lung cancer cells with an* EGFR* mutation [11]. Although acquired resistance cells with increased EGFR phosphorylation (all of them harbored T790M mutation) showed higher PD-L1 expression, osimertinib monotherapy is the current standard treatment for these patients.

Oncogenic signaling within tumor cells and stimuli from the microenvironment both affect PD-L1 expression in tumor cells (constitutive and adaptive PD-L1 expression, resp.). Therefore, our current study looked only at half of the picture (constitutive PD-L1 expression only). However, our results are consistent with clinical findings that showed poorer response rates of PD-1/PD-L1 targeting agents in lung cancer patients with* EGFR* mutations, and may explain at least part of the reasons for the lower efficacy of these agents in these specific patients.

## 5. Conclusions

In summary, we showed that lung cancer cell lines with* EGFR* mutations do not have high PD-L1 protein expression by an FDA approved PD-L1 test. In addition, we demonstrated that PD-L1 expression changes dramatically after acquisition of resistance to EGFR-TKIs, and that was correlated with phosphorylation status of EGFR. Our data implies possible low PD-L1 expression in TKI-refractory lesions without T790M mutation, and that can be one of the molecular mechanisms that attenuates the efficacy of PD-1/PD-L1 targeting agents in lung cancer patients with* EGFR* mutations.
